# Exploration of tongue dorsum sampling to support clinical diagnosis of leprosy patients in the Comoros: A cross-sectional study

**DOI:** 10.1371/journal.pntd.0013541

**Published:** 2025-09-17

**Authors:** Lena Krausser, Maren Van Nieuwenhove, Nissad Attoumani, Silahi H. Grillone, Magalie Van Dyck-Lippens, Leen Rigouts, Abdallah Baco, Wirdane Abdou, Aboubacar Mzembaba, Epco Hasker, Younoussa Assoumani, Bouke C. de Jong, Sofie M. Braet

**Affiliations:** 1 Institute of Tropical Medicine, Antwerp, Belgium; 2 University of Antwerp, Antwerp, Belgium; 3 Research Foundation Flanders, Brussels, Belgium; 4 Damien Foundation, Brussels, Belgium; 5 National Tuberculosis and Leprosy Control Program, Moroni, Comoros; Marie Adelaide Leprosy Centre Pakistan, PAKISTAN

## Abstract

**Background:**

The accuracy of the WHO-endorsed clinical leprosy diagnosis depends on the expertise of health care workers. For molecular confirmation of clinically diagnosed patients, skin biopsies have the highest sensitivity to detect *Mycobacterium leprae*. As less invasive tongue swabs showed promising results for qPCR-based *M. tuberculosis* detection, this study investigated the presence of *M. leprae* on the tongue dorsum of clinically diagnosed leprosy patients.

**Methods and findings:**

During the activities of the (BE-)PEOPLE study, 499 clinically diagnosed, consenting patients from the Comoros were recruited. Samples collected included skin biopsies from active lesions, nasal swabs, tongue swabs, and, in some cases, tongue scrapes. *M. leprae* DNA was quantified with the RLEP qPCR assay and human mitochondrial DNA was quantified as sample adequacy control (SAC).

On 18.1% (90/498) of tongue swabs and 13.2% (12/91) of tongue scrapes *M. leprae* DNA was detected. In only six patients tongue scrapes outperformed the tongue swab based on the number of bacilli/sample. Except for two paucibacillary (PB) patients, all 100/102 positive tongue samples were from multibacillary (MB) patients. Only patients with a RLEP-positive skin biopsy and positive bacteriological index (BI) yielded *M. leprae* DNA on the tongue scrape. The skin biopsy samples had a sensitivity of 92.5% (248/268) for MB and 74.3% (130/175) for paucibacillary (PB) patients. Nasal swabs were positive for 60.2% (162/269) of MB but only 2.2% (5/229) of PB patients.

**Conclusion:**

This is the first study to identify *M. leprae* bacilli on the tongue dorsum of clinically diagnosed leprosy patients by RLEP qPCR. Due to low positivity rates, tongue sampling has limited added value over skin biopsies and nasal swabs for the microbiological confirmation of leprosy. However, the mouth in general and the tongue specifically remain interesting sampling sites to gain further insights on the distribution of *M. leprae* bacilli in the body and potential transmission modes.

## Author summary

Leprosy, the oldest infectious disease known to humankind, remains endemic in certain regions of the world. The diagnosis endorsed by the WHO is solely based on the evaluation of clinical signs. It is possible to detect *Mycobacterium leprae* DNA in invasive skin biopsies from lesions for the microbiological confirmation of clinically diagnosed patients. In the search of a less invasive sample, we evaluated tongue swabbing and scraping. This study demonstrated, by using RLEP qPCR, that *M. leprae* DNA can be detected on the tongue of patients with a high bacterial load in their skin biopsies. Further, nasal swabs also showed a higher sensitivity compared to tongue samples, with a larger number of patients testing positive through nasal swabbing. Therefore, the different tongue samplings (both swabs and scrapes) do not have additional value to skin sampling for the molecular diagnosis of leprosy. However, as for the first time *M. leprae* was detected with a DNA-based assay on tongue swabs and scrapes, future research of this sampling site has the potential to give further insights into leprosy transmission.

## Introduction

Leprosy, an infectious disease caused by *Mycobacterium leprae* and to a lesser extent *Mycobacterium lepromatosis* [1], mainly affects the skin and peripheral nerves. The WHO-recommended diagnosis is based on clinical signs only and classifies patients as paucibacillary (PB) and multibacillary (MB) based on the number of skin lesions and presence of acid-fast bacilli in a microscopy smear. Patients with more than five lesions and/or a positive smear are classified as MB [2].

Although currently not endorsed by WHO, molecular methods detecting *M. leprae* DNA in patient samples are more objective than visually evaluating clinical signs. However, their limit of detection does not allow confirmation of all PB patients. *M. leprae* DNA has been detected in nasal swabs [3–5], blood [6,7], and urine [8,9], but skin biopsies yield the highest sensitivity. For example, with the RLEP qPCR, targeting 36 copies of the *M. leprae* specific repetitive element, *M. leprae* DNA was detected in the skin of 80.1 to 98.7% of MB and 61.7 to 67.5% of PB patients [3,10]. Also, household contacts of both PB and MB patients may carry *M. leprae* DNA in their nose [4].

Oral sampling for the molecular confirmation of leprosy has been investigated in five studies to date, only including patients from Brazil. However, sampling sites and targets for the molecular confirmation differed. In 2011, Martinez *et al.* were able to detect *M. leprae* DNA on buccal swabs by RLEP PCR in 20.2% of MB and 12.4% of PB patients [11]. Using a single-copy *mntH* PCR de Abreu *et al.* confirmed the presence of *M. leprae* DNA in oral mucosa biopsies, with a sensitivity of 60.6% in the tongue mucosa [12]. They also confirmed the presence of free *M. leprae* bacilli on the tongue by immunohistochemistry [12]. In saliva samples, 38.5% of MB and 31.6% of PB patients were positive by a qPCR targeting the 85A-C intergenic region [13]. More invasive palate mucosa scraping yielded positivity rates of 36.0% for MB and 33.0% for PB patients by RLEP qPCR [14]. Most recently, Manta *et al.* found that buccal swabs are positive on 16S rRNA qPCR in 13.0% of patients, regardless of clinical classification [5]. To date, the presence of *M. leprae* on the tongue dorsum has not been investigated systematically with molecular methods. Unlike for *M. tuberculosis* which spreads to the tongue through the passage of infected sputum during expectoration, the mechanism how *M. leprae* may spread to the tongue dorsum are no elucidated yet. For the diagnosis of tuberculosis (TB), qPCR on tongue swab extracts for the repetitive IS*6110* target reached a sensitivity of around 90% compared to GeneXpert testing on sputum as gold standard [15], suggesting it as a promising sampling method. As the yield of general bacterial biomass does not diminish with repeated swabbing, pooling of tongue swabs has been proposed to increase sensitivity [15].

For swabs it is often visually unclear whether sampling was performed according to protocol. During the COVID-19 pandemic sample adequacy controls (SAC) were introduced to monitor self-testing [16]. These quantify human DNA targets, like RNase P, to confirm sample quality and reduce false negatives [16,17]. In the study proposing tongue swabbing for TB diagnosis, a mitochondrial DNA (mtDNA) qPCR was able to distinguish oral samples from control samples from the hand [18]. Further, a sample processing control (SPC), non-target DNA spiked into samples and detected by a separate primer/probe set, can assess amplification efficiency and inhibition [19]. However, quantification may be distorted in samples with a high target burden through reagent competition or fluorescent spillover.

To date, the Union of the Comoros remains one of the 23 WHO global priority countries for leprosy with a persistently high new case detection rate of 3.4/10,000 in 2024 [20]. On Anjouan, the most affected island, and Mohéli all clinically diagnosed leprosy patients are offered enrolment in a substudy of the (BE-)PEOPLE study, a randomised controlled trial assessing the influence of post-exposure prophylaxis. In this study we comprehensively compare four different sample types - skin biopsies, nasal swabs, tongue swabs and tongue scrapes - with regards to *M. leprae* DNA quantity. A qPCR assay targeting human mtDNA in all swab-based samples allowed to assess the sampling quality. The aim was to investigate the presence and quantity of *M. leprae* on the tongue dorsum and to assess whether this minimally invasive sampling site could provide added diagnostic value compared to skin biopsies and nasal swabs.

## Materials and methods

### Ethics statement

The protocols of the PEOPLE (Clinicaltrials.gov NCT03662022) and BE-PEOPLE studies (Clinicaltrials.gov: NCT05406479) were approved by the Institutional Review Board of the Institute of Tropical Medicine (ITM), Antwerp, Belgium, the Ethics Committee of the University Hospital, Antwerp, and the National Ethics Committee of the Union of the Comoros, Moroni, Grande Comore.

### Patients

**Fig 1:**
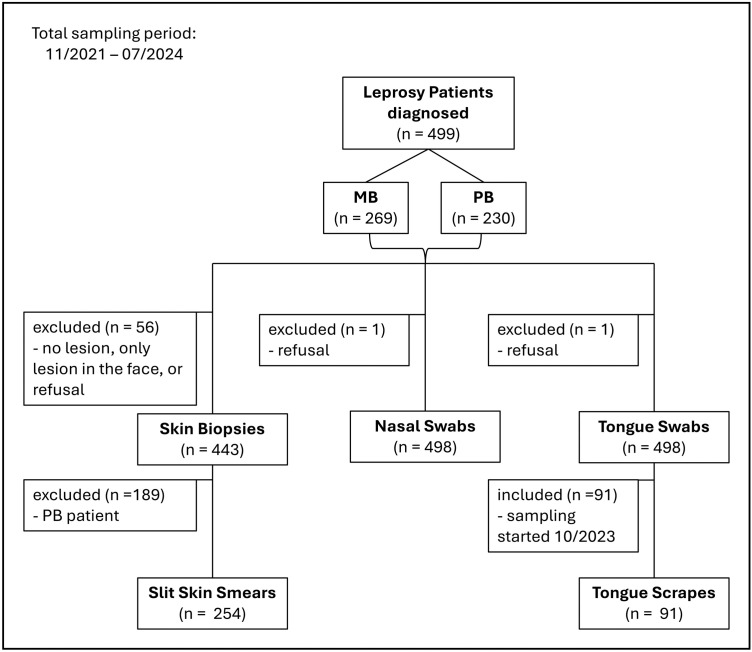
Leprosy patients diagnosed in the (BE-)-PEOPLE studies between 11/2021 and 07/2024. Only leprosy patients diagnosed according to WHO criteria were included in this study. Patients could selectively refuse the collection of specific sample types. Patients without lesions or only lesions in the face were not included for skin biopsy sampling. The collection of tongue scrapes only started in 10/2023. For patients included after this date, tongue scraping was always performed after tongue swabbing. MB: multibacillary, PB: paucibacillary.

All leprosy patients diagnosed according to WHO guidelines during annual door-to-door screenings on Anjouan and Mohéli were eligible to participate in this substudy of the PEOPLE and BE-PEOPLE studies. This analysis includes n = 499 patients who were asked to participate in the studies between November 2021 and July 2024 (Fig 1). Each participant or parent/guardian of minors gave written informed consent. From minors (12 – 17 years) also signed assent was obtained.

### Sample collection

All sample types were collected after clinical leprosy diagnosis and informed consent (Fig 1). Exclusion criteria for sampling were patient refusal, and additionally for skin biopsy sampling the absence of lesions and presence of lesions only in the face. A 4-mm skin biopsy was taken from the edge of a lesion under local anaesthesia (n = 443). For nasal swabs a FLOQSwab (Copan, Italy) was rubbed against the inner walls of both nostrils (n = 498). Tongue swabs were collected by rolling/rubbing the swab on the front 2/3rds of the tongue dorsum for 15 seconds (n = 498). After tongue swabbing, more biofilm was collected with plastic tongue scrapers used for oral hygiene [21] and transferred to a tube using a clean swab. This study includes patients diagnosed from November 2021; however, tongue scrape sampling was only introduced in October 2023. Therefore, this sample type is only available for a subset of patients (n = 91). Additionally, a slit skin smear (SSS) was performed from the lesion where the skin biopsy was collected to determine the bacteriological index (BI) for MB patients (n = 254). Environmental controls were taken on each sampling day by exposing a swab to the air for 1 min in the room where the sampling took place. All samples except for SSS were submerged in disolol (ethanol denatured with 1% isopropanol and 1% methyl ethyl ketone) and stored at -20 °C before shipment to the ITM at ambient temperature where DNA extractions were performed.

### DNA extraction

A sample processing control (SPC, Eurogentec, Belgium) was added to samples before processing. For swabs, disolol was removed by centrifugation and sedimented material was concentrated in 200 µl phosphate-buffered saline (PBS). Skin biopsies were halved, rehydrated in PBS, and homogenized with the gentleMACS dissociator in M tubes (Miltenyi Biotec, Germany). For lysis, 200 µl of the sample suspensions were treated with an in-house lysis buffer (1.6 M GuHCl, 60 mM Tris pH 7.5, 1%, Triton X-100, 60 mM EDTA, 10% Tween-20) and proteinase K (20 mg/ml) for one hour or up to overnight at 60 °C and DNA was purified with the Maxwell RSC DNA FFPE kit (AS1450, Promega, USA) [3].

### qPCR assays

For qPCR assays, 2 µl DNA extract was mixed with 18 µl master mix including Sensimix II (Eurogentec, Belgium). The RLEP fragment was amplified with a primer and probe set described previously [22] at 95 °C for 10 min followed by 45 cycles of 10 sec at 95 °C and 1 min at 60 °C on a StepOnePlus instrument (Applied Biosystems, USA). Human mtDNA qPCRs were run with a primer and probe set described previously [23] at 95 °C for 10 min followed by 45 cycles of 10 sec at 95 °C and 2 min at 64 °C. Each run included negative controls (nuclease-free water) and a standard curve of target-specific plasmid DNA (TIB MOLBIOL, Germany) for absolute quantification, with the Y-intercept (~ C_q_ 40) serving as zero value. All samples were tested with a positivity cutoff of C_q_ < 40 in minimal two of three replicates, based on previous publications to detect even small quantities of *M. leprae* DNA [3,10,14].

### Statistical analysis

The sample size calculation for this cross-sectional study was performed using the following formula [24]:


$$Sample\;size=\frac{{\left(Z_{\text{1}-\frac{\alpha }{\text{2}}}\right)}^{\text{2}}(p)(q)}{d^{\text{2}}},$$

with $Z_{\text{1}-\frac{\alpha }{\text{2}}}$ = 1.96, p = previously reported sensitivity, q = 1 – p, d = 0.05. Based on a previous study the sensitivity of the different sample types was assumed at for 80.1% for skin biopsies and 25.7% for nasal swabs [3]. As this is the first study specifically testing the tongue dorsum, the theoretical sensitivity was set at 50%.

Quantities of the multi-copy target RLEP were extrapolated to the input sample and translated to bacilli per sample. To each bacillary quantity 1 was added to avoid zero values for the log10-transformation.


$$\begin{array}{c} bacilli\;per\;biopsy={log}_{\text{1}\text{0}}\left(\text{1}+\left(\frac{bacilli\;per\;\text{2}\;\mu l}{\text{3}\text{6}\;RLEP\;copies}\;\times \text{1}\text{2}\text{5}\right)\right) \end{array}$$

$$\begin{array}{c} bacilli\;per\;swab={log}_{\text{1}\text{0}}\left(\text{1}+\left(\frac{bacilli\;per\;\text{2}\;\mu l}{\text{3}\text{6}\;RLEP\;copies}\;\times \text{2}\text{5}\right)\right) \end{array}$$

For mtDNA, the quantity was extrapolated to the whole sample and log10 transformed. SPC results were analysed based on the raw C_q_-values. The differences in RLEP qPCR positivity across all sample types were analysed with pairwise McNemar’s tests and a Bonferroni correction was applied to account for the pairwise comparisons. Only patients with results available for both samples in each comparison were included. To assess the differences in sensitivity of the RLEP assay for MB and PB patients, the Chi-Square test with Yates’ continuity correction was used. Spearman’s rank correlation coefficient ρ was calculated to assess the correlation between the quantities of bacilli found in the different sample types [25]. To analyse the difference between the mtDNA quantities and SPC C_q_-values in different samples, Wilcoxon rank sum tests with continuity correction and pairwise Wilcoxon Rank-Sum tests were performed. For all statistical tests the alternative hypothesis was accepted at a significance level α = 0.05. Analyses were performed with R version 4.4.3 (The R Foundation, Austria).

## Results

### Clinical diagnosis and sample size calculation

A total of 499 patients were recruited from November 2021 to July 2024 of which n = 269 were diagnosed as MB and n = 230 as PB. Skin biopsies were collected from n = 443 and tongue scrapes from n = 91 patients. All except for one patient provided nasal and tongue swabs. Of the n = 269 MB patients, n = 254 provided a SSS and n = 142 had a positive BI.

The sample size calculations resulted in n = 245 skin biopsies and n = 293 nasal swabs and n = 384 tongue swabs and scrapes. Accordingly, the collected number of samples was sufficient for all samples except for tongue scrapes.

### Environmental, extraction, and qPCR controls confirm validity of qPCR results

All environmental controls, negative extraction controls and negative qPCR controls were negative, making environmental and cross-sample contamination very unlikely. Positive extraction and qPCR controls yielded the expected results. Therefore, all qPCR results used in the analyses are considered valid, no results were removed from the analysis.

### RLEP qPCR positivity remains highest in skin biopsies from MB patients

With the RLEP qPCR, the overall sensitivity is highest in skin biopsies reaching 85.3% (Table 1). To assess the difference in positivity of the RLEP qPCR across sample types, a pairwise comparison was performed between all sample types. All comparisons except between tongue swabs and scrapes were significant (Table 2), confirming that the positivity is highest in skin biopsies, followed by nasal swabs and tongue samples.

**Table 1:** Positivity rates of the RLEP qPCR assay in different sample types.

	Overall positivity		MB								PB	
			BI = 0		BI > 0		BI = NA		Total			
Skin biopsy	85.3% (CI: 81.7 – 88.5%)	378/443^1^	83.0% (CI: 74.8 – 89.5%)	93/112	100% (CI: 97.4 – 100%)	142/142	92.9% (CI: 66.1 – 99.8%)	13/14	92.5% (CI: 88.7 – 95.4%	248/268	74.3% (CI: 67.1 – 80.6%)	130/175
Nasal swab	33.5% (CI: 29.4 – 37.9%)	167/498	24.1% (CI: 16.5 – 33.1%)	27/112	90.1% (CI: 84.0 – 94.5%)	128/142	46.7% (CI: 21.3 – 73.4%)	7/15	60.2% (CI: 54.1 – 66.1%)	162/269	2.2% (CI: 0.71 – 5.0%)	5/229
Tongue swab	18.1% (CI: 14.8 – 21.7%)	90/498	4.5% (CI: 1.5 – 10.1%)	5/112	56.3% (CI: 47.8 – 64.6%)	80/142	20.0% (CI: 4.3 – 48.1%)	3/15	32.7% (CI: 27.1 – 38.7%)	88/269	0.87% (CI: 0.11 – 3.1%)	2/229
Tongue scrape	13.2% (CI: 7.0 – 21.9%)	12/91^2^	0% (CI: 0 – 19.5%)	0/17	41.4% (CI: 23.5 – 61.1%)	12/29	0% (CI: 0 – 60.2%)	0/4	24.0% (CI: 13.1 – 38.2%)	12/50	0% (CI: 0 – 8.6%)	0/41

^1^Patients only provided a skin biopsy when a lesion was present and not in the face.

^2^Tongue scrapes are only available for a subset of patients because collection was introduced later.

MB: multibacillary, PB: paucibacillary, BI: bacteriological index, NA: not available, CI: 95% confidence interval

**Table 2:** Pairwise comparisons of positivity of the RLEP qPCR across sample types.

Comparison of sample types	Number of patients	Adjusted p-value*
Skin biopsy vs. nasal swab	442	< 0.0001
Skin biopsy vs. tongue swab	442	< 0.0001
Skin biopsy vs. tongue scrape	78	< 0.0001
Nasal swab vs. tongue swab	498	< 0.0001
Nasal swab vs. tongue scrape	91	< 0.0001
Tongue swab vs. tongue scrape	91	1.00

*p-values were adjusted with a Bonferroni correction to account for multiple comparisons

**Fig 2:**
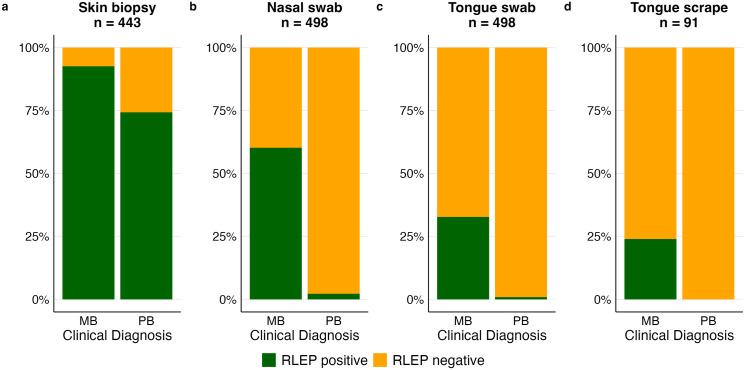
Yield of positive RLEP qPCR results over different sample types. The proportion of positive RLEP qPCR results was calculated per sample type and type of clinical diagnosis. For skin biopsies and tongue scrapes the results only represent the subset of patients for which the samples were available. The cutoff for positivity was set at a C_q_-value of 40. MB: multibacillary, PB: paucibacillary.

A stratification by clinical diagnosis showed that for all sample types the proportion of positive results was significantly higher in MB than in PB patients (Table 1 and Fig 2). Of the available skin biopsies, 92.5% of the MB patient samples and 74.3% of PB patient samples were positive (p < 0.001). Nasal swabs were positive in 60.2% of MB and 2.2% of PB patients (p < 0.001), tongue swabs in 32.7% of MB patients and 0.87% of PB patients (p < 0.001). For tongue scrapes, no PB patient had a positive result while 24.0% of MB patients harboured *M. leprae* DNA (p = 0.002), however only for BI-positive patients. Of patients who did not provide a skin biopsy, only two had a positive result on a different sample type (nasal swab) and of patients who were RLEP-negative on the skin biopsy, only one had a positive result in one of the other samples (nasal swab).

### Correlation of bacterial load between the sample types

For an estimation of the relationship between the qPCR-derived bacterial load on different sample types from a patient, Spearman’s rank correlation test between the qPCR-derived bacterial load on skin biopsies and nasal swabs resulted in a coefficient ρ = 0.79 for skin biopsies and nasal swabs, indicating a strong correlation [25]. For tongue swabs and scrapes this value was lower with ρ = 0.61 and ρ = 0.54, respectively, indicating a moderate correlation with the bacterial load on skin biopsies.

**Fig 3:**
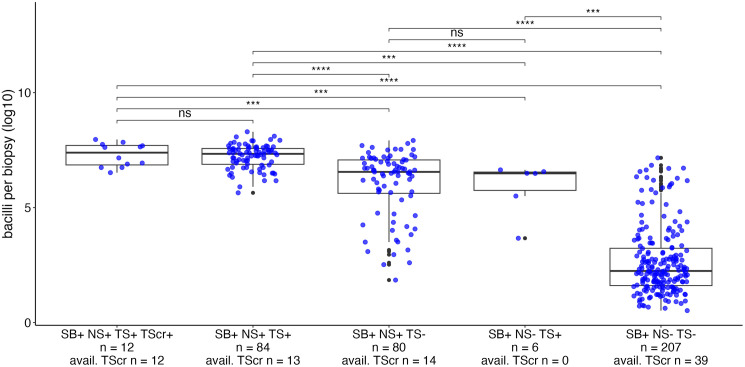
Median quantity of bacilli in the skin biopsies of patients belonging to different groups. Boxplots depict the median and interquartile range of the log10-transformed bacterial load on the respective skin biopsies of patients belonging to the groups indicated on the x-axis. The results of a pairwise Wilcoxon Rank-Sum test are depicted, ****: p-value < 0.0001, ***: p-value < 0.001, ns: no significant. SB: skin biopsy, NS: nasal swab, TS: tongue swab, TScr: tongue scrape, + /-: positive/negative RLEP qPCR for respective sample.

Subsequently, the bacterial load in different sampling sites was assessed for different patient groups. All of the 12 patients with a positive tongue scrape were positive for all other samples and exhibited high bacterial loads in their skin biopsies ranging from 3.31 x 10^6^ to 9.15 x 10^7^ bacilli/biopsy (Fig 3). Of the ten patients with a negative tongue scrape and the highest bacterial loads in the skin biopsies, the qPCR of the tongue scrape remained negative also after a 1:10 dilution, ruling out the influence of qPCR inhibitors [26]. Tongue swabs were positive for 90 patients, who all also had positive skin biopsy results with an estimated 4.65 x 10^3^ to 1.98 x 10^8^ bacilli/biopsy. Six of these 90 patients had an RLEP-negative nasal swab, reflecting patients with only up to 4.36 x 10^6^ bacilli/biopsy. Overall, patients with higher bacterial loads on the skin biopsy tended to also be positive on a different sample (Fig 3).

Further, the number of bacilli in skin biopsies and nasal swabs stratified by qPCR results of the tongue swab was analysed. For patients who had a positive RLEP result on their tongue swab, the number of bacilli in the skin biopsy was significantly higher with a median of 1.97 x 10^7^/biopsy (5.52 x 10^6^ – 3.70 x 10^7^) compared to a median of 217.84/biopsy (15.46 – 4.53 x 10^4^) for patients with a negative tongue swab (p < 0.001). Similarly, the number of bacilli on the nasal swabs was higher for patients with a positive tongue swab result, with a median of 523.74 bacilli/nasal swab (145.44 -2697.30), than for patients with a negative tongue swab (Table 3).

**Table 3:** Number of bacilli on skin biopsies and nasal swabs defined by RLEP qPCR, stratified by tongue swab positivity.

	RLEP qPCR result tongue swab (n)	Median number bacilli/sample	IQR	p-value
Skin biopsy	Positive (90)	1.97 x 10^7^	5.52 x 10^6^ – 3.70 x 10^7^	<2.2e-16
	Negative (352)	217.84	15.46 – 4.53 x 10^4^	
Nasal swab	Positive (90)	523.74	145.44 -2697.30	<2.2e-16
	Negative (408)	0	0	

IQR: interquartile range

### Human mtDNA assay

**Fig 4:**
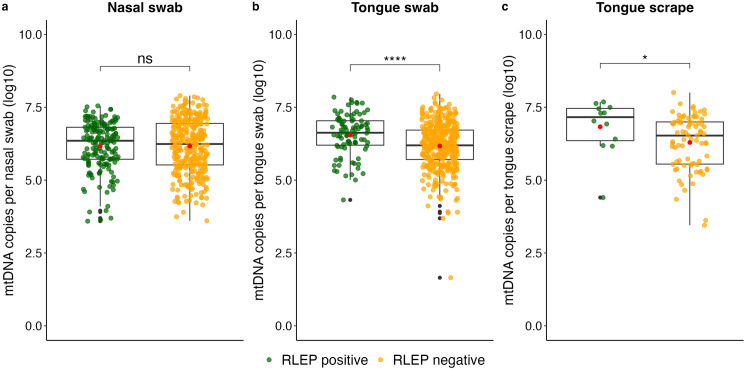
Quantity of human mtDNA detected in swab-based samples. Median mtDNA copies per samples type are depicted, stratified by positivity on the respective sample. Boxplots show the median of the log10-transformed mtDNA quantity per swab with interquartile range; black dots represent outliers. The results of a Wilcoxon Rank-Sum test are depicted, with ****: p-value < 0.0001, *: p-value < 0.05, ns: no significant. The red dot indicates the mean.

For all swab-based samples also human DNA was quantified to ascertain that the swab had been in contact with the sampling site. All swabs yielded a positive result for the detection of human DNA. Stratified for RLEP positivity, no significant difference in the mtDNA quantity was detected in nasal swabs. However, the median mtDNA quantity was significantly lower for RLEP-negative compared to RLEP-positive tongue swabs (p < 0.001) and scrapes (p < 0.001) (Fig 4). Nevertheless, on average the same quantity of mtDNA was recovered from nasal swabs, tongue swabs and scrapes (Fig 4). Overall, six patients had a negative RLEP qPCR result for the tongue swab with an mtDNA qPCR result classified as an outlier, indicating that the quantity of human DNA collected was very low (Fig 4).

### Sample processing control assay

**Fig 5:**
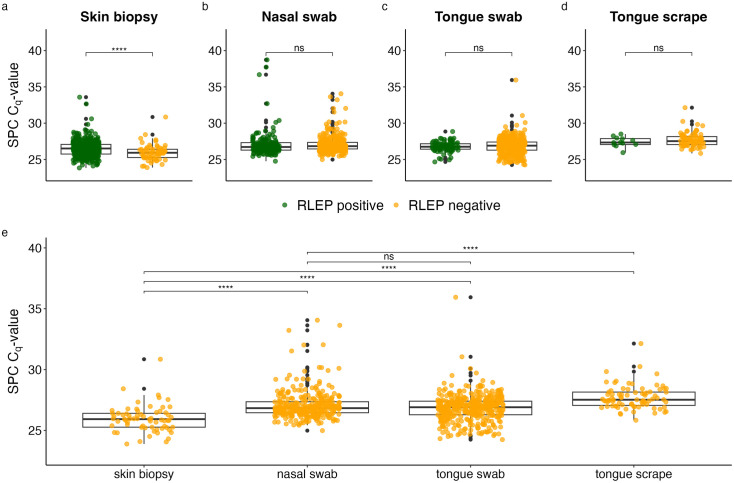
C_q_-values of SPC measured in the different sample types. The plots in the top row (a - d) depict the C_q_-values stratified by RLEP positivity in the sample. The plot on the bottom (e) depicts only the samples with a negative RLEP qPCR result. Boxplots show the median of the SPC C_q_-values with interquartile range; black dots represent outliers. The results of a Wilcoxon Rank-Sum test are depicted for the skin biopsies, ****: p-value < 0.0001, ns: not significant.

The SPC controls were included to estimate potential inhibition during extraction and qPCR. The SPC C_q_-values stratified by RLEP positivity were significantly higher for skin biopsies with a positive RLEP result compared to those where no *M. leprae* DNA was detected (p < 0.001), suggesting inhibition or competition for the RLEP-negative samples (Fig 5). For the other sample types this difference was not significant. A pairwise analysis of RLEP-negative samples showed that the SPC C_q_-values for nasal and tongue swabs are not significantly different, while for skin biopsies the SPC C_q_-values were lower than for all the other samples (Fig 5). To investigate the potential influence of the quantity of human DNA on the SPC results, a Spearman correlation analysis of the SPC C_q_-value and the number of mtDNA copies in all swabs was performed. It showed a moderate inverse correlation for tongue scrapes (ρ = -0.44) but only a weak correlation for tongue and nasal swabs.

## Discussion

Based on promising findings of tongue swab sampling for the molecular confirmation of TB, and previous reports of *M. leprae* carriage on the tongue of leprosy patients, this study analysed the presence of *M. leprae* DNA on tongue swabs and scrapes compared to skin biopsies and nasal swabs. For the first time, we confirmed the presence of *M. leprae* DNA on tongue swabs for 32.7% MB and 0.87% PB patients with the sensitive RLEP qPCR assay. Other studies found *M. leprae* DNA at similar rates in in superficial oral samples like buccal swabs and saliva [5,11,13]. Nevertheless, more invasive sampling like oral skin slit scraping and biopsies resulted in higher positivity rates between 33.0% and 69.7% in previous studies [12,14]. Therefore, aiming to collect more biofilm from deeper between the tongue papillae, our study also tested scraping the tongue with minimally invasive plastic tongue scrapers used for oral hygiene. With these, we detected *M. leprae* DNA in only 24.0% of MB patients, making it less sensitive than swabbing.

Potential mechanisms contributing to the lower-than-expected sensitivity in tongue scrapes are qPCR inhibition and inadequate sampling. Like saliva, biomass collected from tongue swabs and scrapers can contain qPCR inhibitors, such as food particles, digestive enzymes, and mucins [27,28]. For RLEP-negative samples from the tested cohort, results of the sample processing controls suggested that the influence of qPCR inhibition is highest in tongue scrapes. Also, unexpectedly, tongue scrapers did not collect more human material than swabs. Additionally, for both tongue sampling methods less human DNA was detected in RLEP-negative samples. Therefore, insufficiently applied pressure during sampling could have played a role in the lower RLEP positivity for any type of tongue sample. Finally, the decision to perform subsequent sampling of tongue swabs and scrapes rather than randomising the sampling order in our study may have impaired the comparison, as swabbing may have already collected most of the mycobacterial material on the tongue. Although a previous study indicated that repeated swabbing does not decrease the general bacterial load on the tongue [15], this may not be the case for *M. leprae*.

Importantly, tongue scraping was introduced later into the sampling workflow, and the smaller sample size limits statistical power. Although, the findings should be interpreted with caution, the observed trends do not support tongue scraping as having an additional value over tongue swabs and the other sample types.

Overall, with around 60% in a previous study [12] the sensitivity of detecting *M. leprae* DNA in tongue biopsy tissue is higher than by swabbing or scraping the tongue dorsum. This indicates that unlike in tissue, the superficial carriage of *M. leprae* DNA on the tongue may be of transient nature.

The mechanisms how *M. leprae* may migrate to the tongue dorsum are still speculative. One hypothesis would be via oral lesions that have mainly been reported for MB or lepromatous leprosy patients and can contain acid-fast bacilli [29,30]. Here, most commonly the hard palate is affected, probably because of the lower temperature, favouring *M. leprae* survival [30]. *M. leprae* may therefore be transferred to the tongue dorsum by rubbing the hard palate or other oral lesions. Although we found *M. leprae* DNA on the tongue of MB patients with high bacterial loads in their skin biopsies, none of the patients had recorded oral leprosy lesions, potentially explaining the low overall proportion of positive tongue samples. This could be a result of the efficient control programme in the Comoros, preventing high rates of grade 2 disabilities and potentially also oral lesions. An alternative hypothesis could be the dissemination of *M. leprae* from the nose to the tongue dorsum via infected nasal secretions that pass through the choanae, small openings connecting the nasal cavity with the nasopharynx and the mouth. The carriage of *M. leprae* DNA in the nose was confirmed in 60.2% of the MB and 2.2% of the PB patients, in line with previous reports of *M. leprae* bacilli in nasal secretions [31,32]. This hypothesis would also explain the absence of RLEP positivity on tongue samples in PB patients who often do not carry *M. leprae* DNA in their nose. Of note, six patients with a positive tongue swab had a negative nasal swab, either indicating that the *M. leprae* load in the nose was near the limit of detection or that the mouth could harbour *M. leprae* independently from nasal secretions. However, overall the sampling of the tongue dorsum with swabs and scrapers yielded significantly lower RLEP qPCR positivity rates compared to nasal swabbing, making nasal swabs the superior minimally invasive sample.

Although the detection of DNA alone does not allow to conclude whether the genetic material stems from free DNA, viable or dead bacilli, and no viability testing was performed, it can be speculated that the oral cavity has a potential role in the transmission of leprosy, e.g., by shedding bacilli and transporting them in droplets when speaking.

In our study, skin biopsies yielded most *M. leprae* DNA, in line with findings of other studies [3,10]. The positivity of 92.5% in MB and 74.3% in PB patients is higher than in a previous study from the Comoros [3] and could be the result of optimised DNA extraction and more refined clinical diagnosis of the experienced health care workers during active screenings. Although the C_q_-cutoff used for the RLEP qPCR varies between studies, 40 C_q_ as a positivity cutoff has been applied by several studies [3,10,14]. This cutoff was selected to be able to detect the minute quantities of *M. leprae* DNA that are sometimes present especially in PB samples, while controlling for contamination. Although no healthy controls were included, all environmental controls were negative for RLEP, indicating a low risk for sample contamination. RLEP-positive skin biopsies had on average significantly higher C_q_-values for the sample processing controls than RLEP-negative ones, suggesting competition for amplification reagents in cases of highly RLEP-positive samples [33]. Of the 56 patients who did not provide a skin biopsy, none showed positivity in either of the tongue samples. Therefore, together with the general low sensitivity, tongue swabs and scrapes have a limited added value for molecular confirmation of clinical leprosy diagnosis compared to skin biopsies and nasal swabs. However, RLEP positivity on the tongue can indicate a high bacterial load patient, similar to nasal swabs in Braet *et al*. [3].

This study showed for the first time on a large patient cohort that *M. leprae* DNA can be detected on the tongue dorsum of MB leprosy patients with a high bacterial load. Given that other minimally invasive sampling types have a lower sensitivity than skin biopsies, the lower yield of *M. leprae* DNA on the tongue is not surprising. Due to the very low positivity rate of tongue samples, it is not advised to include tongue sampling in routine diagnostic testing or epidemiological surveillance. However, investigating this sampling site in populations with higher rates of oral lesions [29] could be informative on the *M. leprae* oral carriage and potentially to investigate intra-patient *M. leprae* strain variability. Further, viability assays [34] and tongue testing in household contacts could give valuable insights on the migration of *M. leprae* within the body and has the potential to contribute to resolving the mode transmission of leprosy.
